# Cortical mapping of the infraspinatus muscle in healthy individuals

**DOI:** 10.1186/1471-2202-14-52

**Published:** 2013-04-24

**Authors:** Suzy Ngomo, Catherine Mercier, Jean-Sébastien Roy

**Affiliations:** 1 Department of Rehabilitation, Faculty of Medicine, Laval University, Quebec City, QC, G1R 1P5, Canada; 2 Centre for Interdisciplinary Research in Rehabilitation and Social Integration (CIRRIS), 525, Wilfrid-Hamel Boulevard, suite H-1710, Quebec, QC, G1M 2S8, Canada

**Keywords:** Shoulder, Motor cortex, Motor excitability, Rotator cuff, Motor threshold, Cortical representation, Infraspinatus

## Abstract

**Background:**

While cortical representations of intrinsic hand muscles have been extensively studied in healthy individuals, little is known about the representation of proximal upper limb muscles. Improving our understanding of normal shoulder function is important, given that shoulder musculoskeletal disorders affect approximately 20% of the population and are suspected to involve changes in central motor representations. The purpose of the study is to describe the motor representation (motor evoked potentials (MEP) amplitude at the hotspot, map area, normalized map volume and center of gravity) of the infraspinatus muscle in healthy individuals, and to explore the potential influence of hand dominance on this representation (i.e. symmetry of the excitability and of the location of motor map between sides), as well as the effect of age and gender on motor excitability.

**Results:**

Fifteen healthy participants took part in this study. No significant asymmetry between sides was observed for motor excitability (*p* = 0.14), map area (*p* = 0.73) and normalized map volume (*p* = 0.34). Moreover, no side x intensity interaction was found (*p* = 0.54), indicating similar stimulus response properties. No difference between sides was found in the location of infraspinatus motor representation, either in the mediolateral or anteroposterior axis (*p* > 0.10). Neither age nor gender influenced aMT (*p* > 0.58) or MEP size (*p* > 0.61).

**Conclusions:**

As the cortical representation of infraspinatus muscles was found to be symmetric between sides, both in terms of excitability and location, comparisons between the intact and affected side could be performed in clinical studies, regardless of whether the dominant or non-dominant side is affected. The next step will be to characterize corticospinal excitability and map parameters in populations with shoulder disorders.

## Background

The rotator cuff (RC) is one of the most important muscle groups for shoulder function as it provides dynamic stability at the glenohumeral joint [1]. It is made of four muscles, the supraspinatus, infraspinatus, teres minor and subscapularis. Aside from the infraspinatus, RC muscles are very difficult, if not impossible to access using surface electrodes. Electromyographic (EMG) activity of the infraspinatus muscle can be recorded using surface electrodes over a small window overlying the infraspinatus process where there are no other muscles located between its own muscle tissue and the skin [2]. Brown et al. have shown that EMG signals obtained using skin surface electrodes have a strong correlation with the ones using fine-wire electrodes, showing the validity of surface EMG recording of this muscle [2].

The infraspinatus is a critical muscle for shoulder stability. First, it is one of the primary agonists of glenohumeral lateral rotation. Second, it acts with the subscapularis as humeral head depressors to keep the humeral head centralized within the glenoid fossa during arm elevation. Individuals with shoulder disorders have been known to present alterations in the infraspinatus EMG activity during arm elevation [3,4]. Furthermore, the infraspinatus tendon is one of the most affected following RC tendinopathy [5]. Therefore, the infraspinatus is an essential muscle to assess in populations with impaired shoulder function.

Lately, studies have shown that musculoskeletal (MSK) disorders could be associated with a reorganization of the motor cortex. In fact, changes in the motor representation of key muscles for joint stability such as the transversus abdominis muscle for low back pain or the vastus medialis oblique and vastus lateralis for patellofemoral pain have been demonstrated using transcranial magnetic stimulation (TMS) [6,7]. However, little is known about corticospinal excitability and primary motor cortex (M1) representation of the rotator cuff at the shoulder joint. TMS has been widely used to evaluate the corticospinal projections to upper limb distal muscles, such as the first dorsal interosseus. However, shoulder muscles have received much less attention, with only a few studies investigating proximal muscles such as the deltoid muscle in subjects with RC tears [8], and the lower trapezius muscle in subjects with shoulder instability [9]. In healthy subjects, RC muscles have only been evaluated with TMS in one study that assessed the putative role of the propriospinal system in controlling the infraspinatus muscle [10].

Given that shoulder MSK disorders affect approximately 20% of the population, and that RC muscles are fundamental to normal shoulder function, RC muscles representation in M1 needs to be described in order to establish a basis of comparison for studies in clinical populations. The purpose of the current study is to describe the motor representation of the infraspinatus muscle in healthy individuals, and to explore the potential influence of hand dominance on this representation (i.e. symmetry of the excitability and of the location of the motor map between sides), as well as the effect of age and gender on motor excitability.

## Methods

### Participants

Fifteen healthy participants took part in this study (mean age: 43.9 [standard deviation: 11.5] years, age range: 24 – 63 years; eight men, seven women; thirteen right-handed, two left-handed). Recruitment was intended to secure a range of ages similar to the one of patients with RC tendinopathy [11]. Participants had no history of rheumatoid, inflammatory, degenerative or neurological diseases, as well as no pain or movement limitation to the shoulders, or any history of shoulder surgery or sustained upper extremity MSK disorder. Contraindications for magnetic resonance imaging (MRI) or TMS (e.g. metallic or electronic implants, pregnancy, history of epilepsy, etc.) also constituted exclusion criteria. The Ethics Committee of the Quebec Rehabilitation Institute approved this study and all the participants gave their written consent after being informed of the nature and purpose of the study.

### Study design

Each participant took part in two evaluation sessions within a seven-day period. During the first session, participants completed a questionnaire on sociodemographic data and comorbidities. Then, dominance was determined by the laterality quotient of the revised Edinburgh Handedness Inventory [12]. Afterwards, an anatomical MRI of the brain was obtained to accurately position the coil during cortical mapping with a frameless stereotaxy neuronavigation system (Brainsight, Rogue Research, Montreal, Canada). In the subsequent days, participants took part in a second evaluation session during which cortical mapping of M1 for infraspinatus muscle was performed bilaterally.

### Cortical mapping

Cortical motor maps of both infraspinatus muscles were acquired using a Magstim 200 stimulator connected to a 70-mm figure-of-eight coil. Stimuli were applied over grid sites spaced 1 cm apart and positioned over the upper limb area of primary motor cortex of the contralateral hemisphere. Motor evoked potentials (MEPs) were recorded from the EMG recording of the infraspinatus. After skin preparation, a pair of Ag/AgCl surface recording electrodes (1 cm^2^ recording area) was placed over the infraspinatus. A ground electrode was applied on the acromion. Surface electrode placement over the infraspinatus was standardised as described by Delagi & Perotto: 3–4 cm below and running parallel to the spine of the scapula, over the infraspinatus fossa [13]. EMG signals were amplified (1000×), filtered by a band-pass (20–1000 Hz), digitized at a sampling rate of 2000 Hz (Power1401 interface; Cambridge Electronic Design, Cambridge, UK) and stored on a computer for offline analysis. Prior to the experiment, participants were asked to perform isometric maximal voluntary contractions (MVC) in humeral lateral rotation with the shoulder at 0° of elevation and in neutral rotation. Two successive trials were performed with an inter-trial interval of 30 seconds. Maximal value over the two trials was used to compute EMG targets during experimental task (5 ± 1% of MVC). During the mapping procedures, visual feedback of actual EMG activity and of the targeted level of EMG activity was provided in real-time on a screen in front of the participants.

Cortical mapping was performed with the participants in a seated position and actively holding their arm at 45° of humeral abduction (neutral rotation) [14,15]. This arm position brought a light contraction of the infraspinatus corresponding to 5% of MVC. Optimal location for stimulation of the infraspinatus muscle was determined (hotspot) before mapping, as well as the active motor threshold (aMT) at this site. aMT was expressed in percentage of the maximum stimulator output (MSO) and defined as the minimal TMS intensity required to produce discernible MEP amplitudes from the background EMG in at least 50% of the trials (i.e. generating 6 MEPs out of 12 trials) with the infraspinatus slightly contracted (5% ± 1 of MVC). Then, motor mapping was performed using an intensity of stimulation of 110% of aMT (adjusted independently for each side). Six successive pulses separated by intervals of 4.5 to 5 seconds were delivered to each site of the grid [15,16]. A site was considered active if at least two MEPs were elicited. Non-active sites (none or only one MEP) delimited the mapping boundaries. Finally, 12 pulses at 120% and 140% of individual aMT were delivered to the hotspot site to assess corticospinal excitability and to gain some insight into the stimulus–response properties of the infraspinatus muscle. EMG root mean square (RMS) values during the 50-millisecond time windows preceding each TMS pulse were obtained to ensure appropriate contraction levels throughout the experiment (controlled online but also stored for offline quantitative analysis, in order to ensure that any significant difference between sides could not be explained by differences in baseline EMG).

### Data pre-processing and statistical analysis

For each site (as well as for each intensity of stimulation at the hotspot), the peak-to-peak amplitude of the recorded MEP was measured and averaged using custom analysis software (IsotopCM, Mathomic Solutions, Quebec, Canada). The following TMS variables were then extracted: 1) MEP amplitude at the hotspot (at 120% and 140% of aMT), 2) map area, 3) normalized map volume and 4) center of gravity (CoG). Map area was calculated as the sum of the active sites. As standardized grid was used across subjects, the number of active sites truly represents the map area. Normalized map volume was calculated by adding mean amplitudes of each stimulated site divided by the maximum mean amplitude. CoG was computed for the mediolateral (x) and anteroposterior (y) coordinates relative to the vertex (expressed in mm) using the following formula: CoGx = (Σx_i_ * MEP_i_)/ΣMEP_i_ and CoGy = (Σy_i_ * MEP_i_)/ΣMEP_i;_ where MEP_i_ represents the mean amplitude of the MEPs produced at one site.

Descriptive statistics were first calculated for all variables to summarize results. To assess the symmetry of the motor representations, comparisons of aMT and map measures were performed between dominant and non-dominant sides using paired *t*-tests. The symmetry in MEP size (as well as in stimulus–response properties) was assessed using a 2 × 2 repeated measure analysis of variance: intensity of stimulation [120% aMT /140% aMT] X side (dominant [D]/ non-dominant [ND]). Finally, the potential influence of age and gender on motor excitability (aMT and MEP size, dominant side) was assessed, respectively, by Pearson correlations and by paired *t*-tests. All analyses were conducted with SPSS software. Alpha threshold was set at 0.05.

## Results

**Figure 1:**
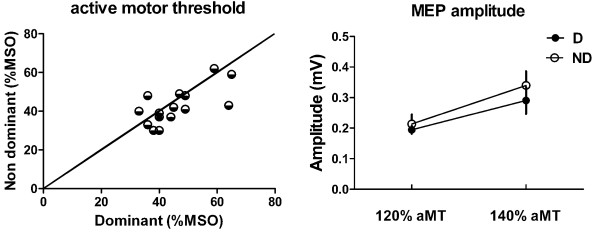
**Comparison of motor excitability between the dominant and non-dominant side. *Left panel*** shows individual results for active motor thresholds (aMT) (expressed in % of maximal stimulator output (MSO)) on both sides. The line of identity, which represents perfect symmetry between sides, is marked. ***Right panel*** shows the average peak-to-peak amplitude of the motor evoked potentials (MEP) for each side, and for each stimulation intensity.

**Figure 2:**
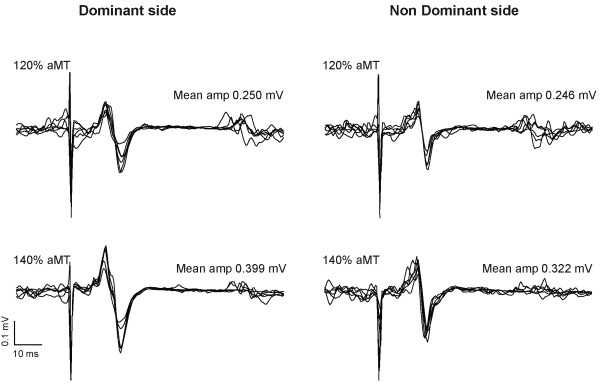
**Example of raw motor evoked potentials obtained in a representative subject.** Six MEPs obtained at the hotspot are shown for each side, and for each stimulation intensity (120% and 140% aMT).

No significant asymmetry in motor excitability was observed between sides. The mean aMT was 45.7 ± 10.1% of MSO for the dominant side, and 42.5 ± 9.4% of MSO for the non-dominant side (*p* = 0.14; Figure 1). As expected, a significant main effect of the intensity of stimulation was found on MEP size (*p* = 0.002), but no effect on the side was observed (*p* = 0.309). Moreover, no side x intensity interaction was found (*p* = 0.539), indicating similar stimulus response properties (within the limits of stimulation intensities tested – see Figure 2 for an example). No significant asymmetry was observed either for the map area (D: 8.6 ± 2.9 cm^2^, ND: 8.1 ± 2.9 cm^2^; *p* = 0.73) or the normalized map volume (D: 6.0 ± 2.5, ND: 5.4 ± 2.2; *p* = 0.34).

**Figure 3:**
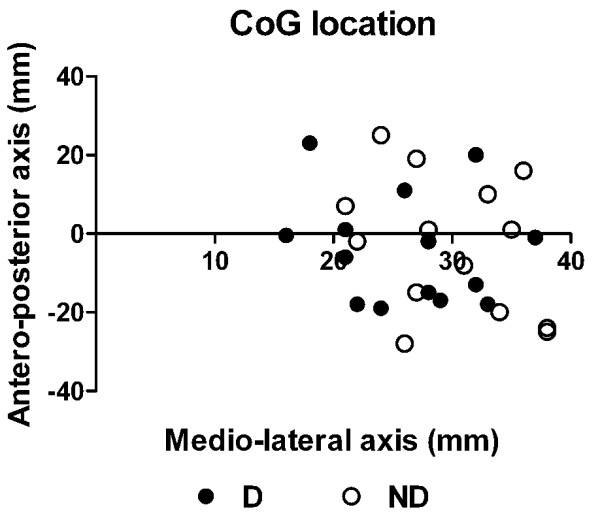
**Comparison of the location of the center of gravity between the dominant and non-dominant side.** The origin is fixed at the intersection between the motor strip and the interhemispheric line. Note that the values on the mediolateral axis have all been converted to positive value (irrespective of the hemisphere tested) to facilitate comparison.

No difference between sides was found in the location of the infraspinatus motor representation, either in the mediolateral (x) or anteroposterior (y) axis (*t* < 0.35; *p* > 0.10; Figure 3). Neither age nor gender influenced aMT (*p* > 0.58; *r* = −0.04 for age; *t* = 0.57 for gender) or MEP size (*p* > 0.28; *r* = −0.23 for age; *t* = 1.13 for gender).

## Discussion

The aim of the present study was to describe the infraspinatus muscle cortical representation, in terms of excitability and location, as well as to assess its symmetry between the dominant and non-dominant sides in healthy individuals. In addition, the influence of age and gender on motor excitability was also explored. Data from healthy males and females from a wide range of ages are reported, which is important to inform future research in populations with impaired shoulder function. Our results on the symmetry of cortical representations indicate no significant difference between the dominant and non-dominant sides. No effect of age or gender was found on motor excitability.

Some authors have reported dominance and age as factors influencing motor excitability [17,18]. However, the evidence on the effect of these variable remains conflicting, with more recent studies showing no impact for these variables, as well as no influence from gender [19,20]. Most importantly, these studies all focused on intrinsic hand muscles. The present results extend these findings by demonstrating that the motor representation of a proximal muscle is similar between sides in healthy individuals, both in terms of excitability and location.

TMS measurements were performed with the muscle slightly contracted (5% MVC). Responses elicited in proximal muscles, such as RC muscles, with the arm at rest are very slight and require high intensities of stimulation [21]. Pilot experiments revealed that it was impossible to map the infraspinatus at rest (and to stimulate at 120 and 140% of resting MT) in several individuals because of high thresholds. It is noteworthy to mention that the posture and level of contraction used for active motor mapping in the present study were determined in order to be easily achievable in most patients with impaired/painful shoulder. A previous study by our research group showed that resting and active cortical maps are similar for hand muscles in healthy individuals, and that both methods provide reliable measures [15].

The infraspinatus is a humeral lateral rotator that has its origins on the infraspinatus fossa of the scapula and its insertion on the greater tubercle of the humerus. One of its primary functions is to depress the humeral head during arm elevation, which prevents subacromial impingement. The infraspinatus is the only RC muscle for which there are no other muscles located between its own muscle tissue and the skin [2]. Therefore, it provides the most direct recording of all RC muscles. EMG activity of the supraspinatus and teres minor could also be evaluated by using surface electrodes, providing the feasibility of evaluating their M1 representations using TMS; however, EMG crosstalk is a bigger issue in this case. The site for recording the supraspinatus using surface electrodes is located over a window where the tendon of the trapezius lies between the muscle and the skin [2]. Therefore, as suggested by Brown et al. [2], the recording may pick up some end-propagating activation from the trapezius. As for the teres minor, it lies deep into the infraspinatus and is assessed from the infraspinatus surface electrode site [2]. Still, Brown et al. [2] have shown that the correlations between skin surface and fine-wire electrodes were high for the supraspinatus and teres minor; however, they were lower than the ones found for the infraspinatus. Possibility of performing cortical mapping of the supraspinatus and teres minor might be explored in the future. Finally, it is not possible to evaluate the subscapularis using surface electrodes as the muscle has its origin from the subscapularis fossa of the scapula, which provides no site for surface electrodes.

Moreover, alterations in infraspinatus muscle activity have been found in populations with shoulder disorders. For instance, in individuals with RC tendinopathy, EMG activity of the infraspinatus has been shown to be significantly decreased between 30 and 60° of shoulder elevation [3]; while during shoulder lateral rotation, significantly less infraspinatus EMG activity was observed on the symptomatic shoulder [4]. Furthermore, a study evaluating the most common location of degenerative RC tears reported that most degenerative cuff tears initiate from a region near the junction of the supraspinatus and infraspinatus tendons [5]. It proves the significant role of the infraspinatus in the stabilization of the glenohumeral joint and in the etiology of chronic RC disorders.

It has been hypothesized that a reorganization of the motor cortex could explain part of the motor control deficits linked to RC disorders [11,22,23]. These central changes could contribute to the chronicity of symptoms. This hypothesis of central changes is based on previous studies that have shown reorganization in the central nervous system in patients with other MSK disorders [24,25]. For example, in patients with patellofemoral pain, On et al. found that the amplitude of MEP produced in quadriceps was significantly increased compared to healthy individuals [6]. Tsao et al. [7] found that the CoG of motor cortical map of the transversus abdominis was more posterior and lateral in patients with recurrent low back pain. Locations of the CoG and map volumes were also correlated with the onset of transversus abdominis EMG during rapid arm movements, suggesting that changes in motor cortical organization could be linked to altered motor control. Therefore, as a fundamental muscle for shoulder stability, better knowledge of the cortical representation of infraspinatus muscle in healthy individuals is important.

Particular concerns for the assessment of changes in cortical representation in MSK disorders affecting the upper limbs relate to the fact that some asymmetry might be related to dominance, an aspect particularly important to control, given that the incidence of RC tendinopathy is higher on the dominant side [11]. The present results suggest that dominance should not be a concern when comparing the motor representation of affected vs. unaffected shoulders in clinical studies. However, this does not indicate that a unilateral MSK disorder cannot affect both hemispheres. An interesting illustration of such bilateral impacts of a MSK lesion was recently provided by Langer et al. [26]. They showed that immobilization (≥14 days) after an upper limb injury resulted not only in a decrease in cortical thickness in the primary motor and somatosensory area corresponding to the injured side, but in an improvement of the motor skills of the non-injured hand that was related to an increase in cortical thickness in the motor cortex corresponding to the non-injured side.

One possible limitation of the present study is that the EMG activity of the infraspinatus was recorded using surface electrodes. The infraspinatus could be seen as a challenging muscle to record with surface electrodes, given that there is only a small window overlying the infraspinatus process where it is possible to directly record EMG activity. Therefore, crosstalk by the trapezius and deltoid is highly probable if the electrode placement is not done accurately. In order to minimize crosstalk, we used standardized procedures for electrode placement that have proven to lead to EMG data in high correlation with data recorded with fine-wire electrodes [2]. Furthermore, verification of electrodes placement and EMG signal quality was done by visual monitoring of signals while subjects performed voluntary contractions. Nevertheless, future studies could validate the present results by using fine-wire electrodes. Finally, the use of surface electrodes simplifies the evaluation of motor representation, which is important to consider if TMS evaluation is to be used in clinical settings.

## Conclusion

The cortical representation of the infraspinatus muscle was found to be symmetrical on either side, both in terms of excitability and location. This suggests that in clinical studies, comparisons between the intact and affected side might be performed, regardless of whether the dominant or non-dominant side is affected. The next step will be to characterize corticospinal excitability and map parameters in populations with RC disorders.

## Abbreviations

aMT: Active motor threshold; CoG: Center of gravity; D: Dominant; EMG: Electromyographic; M1: Primary motor cortex; MEP: Motor evoked potential; MRI: Magnetic resonance imaging; MSO: Maximum stimulator output; MVC: Maximal voluntary contraction; ND: Non-dominant; RC: Rotator cuff; RMS: Root mean square; TMS: Transcranial magnetic stimulation

## Competing interests

The authors declare that they have no competing interests.

## Authors’ contributions

SN: carried out the acquisition and the analysis, participated in the interpretation of data and drafted the manuscript. CM: participated in the design of the study, the analysis and the interpretation of data and drafted the manuscript. JSR: participated in the design of the study, the analysis and the interpretation of data and drafted the manuscript. All authors read and approved the final manuscript.

## References

[B1] Kamkar A Irrgang JJ Whitney SL Nonoperative management of secondary shoulder impingement syndrome J Orthop Sports Phys Ther 1993 17 212 224 8343779 10.2519/jospt.1993.17.5.212

[B2] Brown SH Brookham RL Dickerson CR High-pass filtering surface EMG in an attempt to better represent the signals detected at the intramuscular level Muscle Nerve 2010 41 234 239 19722252 10.1002/mus.21470

[B3] Reddy AS Mohr KJ Pink MM Jobe FW Electromyographic analysis of the deltoid and rotator cuff muscles in persons with subacromial impingement J Shoulder Elbow Surg 2000 9 519 523 10.1067/mse.2000.109410 11155306

[B4] Diederichsen LP Winther A Dyhre-Poulsen P Krogsgaard MR Norregaard J The influence of experimentally induced pain on shoulder muscle activity Exp Brain Res 2009 194 329 337 10.1007/s00221-008-1701-5 19183973

[B5] Kim HM Dahiya N Teefey SA Middleton WD Stobbs G Steger-May K Yamaguchi K Keener JD Location and initiation of degenerative rotator cuff tears: an analysis of three hundred and sixty shoulders J Bone Joint Surg Am 2010 92 1088 1096 10.2106/JBJS.I.00686 20439653 PMC2945926

[B6] On AY Uludag B Taskiran E Ertekin C Differential corticomotor control of a muscle adjacent to a painful joint Neurorehabil Neural Repair 2004 18 127 133 10.1177/0888439004269030 15375272

[B7] Tsao H Galea MP Hodges PW Reorganization of the motor cortex is associated with postural control deficits in recurrent low back pain Brain 2008 131 2161 2171 10.1093/brain/awn154 18669505

[B8] Berth A Pap G Neuman W Awiszus F Central neuromuscular dysfunction of the deltoid muscle in patients with chronic rotator cuff tears J Orthop Traumatol 2009 10 135 141 10.1007/s10195-009-0061-7 19690944 PMC2744738

[B9] Alexander CM Altered control of the trapezius muscle in subjects with non-traumatic shoulder instability Clin Neurophysiol 2007 118 2664 2671 10.1016/j.clinph.2007.09.057 17950033

[B10] Roberts LV Stinear CM Lewis GN Byblow WD Task-dependent modulation of propriospinal inputs to human shoulder J Neurophysiol 2008 100 2109 2114 10.1152/jn.90786.2008 18715892

[B11] Roy JS Moffet H McFadyen BJ Upper limb motor strategies in persons with and without shoulder impingement syndrome across different speeds of movement Clin Biomech 2008 23 1227 1236 10.1016/j.clinbiomech.2008.07.009 18757123

[B12] Oldfield RC The assessment and analysis of handedness: the Edinburgh inventory Neuropsychologia 1971 9 97 113 10.1016/0028-3932(71)90067-4 5146491

[B13] Delagi E Perotto A Anatomic guide for the electromyographer: the Limbs 1980 2 Springfield, IL: Charles C. Thomas

[B14] Gagné M Hétu S Reilly KT Mercier C The map is not the territory: Motor system reorganization in upper limb amputees Hum Brain Mapp 2011 32 509 519 10.1002/hbm.21038 21391244 PMC6870038

[B15] Ngomo S Leonard G Moffet H Mercier C Comparison of transcranial magnetic stimulation measures obtained at rest and under active conditions and their reliability J Neurosci Methods 2011 205 65 71 22227444 10.1016/j.jneumeth.2011.12.012

[B16] Hetu S Gagne M Reilly KT Mercier C Short-term reliability of transcranial magnetic stimulation motor maps in upper limb amputees J Clin Neurosci 2011 18 728 730 10.1016/j.jocn.2010.09.011 21393001

[B17] Triggs WJ Calvanio R Macdonell RA Cros D Chiappa KH Physiological motor asymmetry in human handedness: evidence from transcranial magnetic stimulation Brain Res 1994 636 270 276 10.1016/0006-8993(94)91026-X 8012811

[B18] Matsunaga K Uozumi T Tsuji S Murai Y Age-dependent changes in physiological threshold asymmetries for the motor evoked potential and silent period following transcranial magnetic stimulation Electroencephalogr Clin Neurophysiol 1998 109 502 507 10.1016/S1388-2457(98)00020-0 10030682

[B19] Smith AE Sale MV Higgins RD Wittert GA Pitcher JB Male human motor cortex stimulus–response characteristics are not altered by aging J Appl Physiol 2011 110 206 212 10.1152/japplphysiol.00403.2010 21071590

[B20] Livingston SC Goodkin HP Ingersoll CD The influence of gender, hand dominance, and upper extremity length on motor evoked potentials J Clin Monit Comput 2010 24 427 436 10.1007/s10877-010-9267-8 21110222

[B21] Rothwell JC Thompson PD Day BL Boyd S Marsden CD Stimulation of the human motor cortex through the scalp Exp Physiol 1991 76 159 200 2059424 10.1113/expphysiol.1991.sp003485

[B22] Roy JS Moffet H Hebert LJ Lirette R Effect of motor control and strengthening exercises on shoulder function in persons with impingement syndrome: a single-subject study design Man Ther 2009 14 180 188 10.1016/j.math.2008.01.010 18358760

[B23] van Vliet PM Heneghan NR Motor control and the management of musculoskeletal dysfunction Man Ther 2006 11 208 213 10.1016/j.math.2006.03.009 16781184

[B24] Cowan SM Bennell KL Hodges PW Crossley KM McConnell J Simultaneous feedforward recruitment of the vasti in untrained postural tasks can be restored by physical therapy J Orthop Res 2003 21 553 558 10.1016/S0736-0266(02)00191-2 12706031

[B25] Tsao H Hodges PW Immediate changes in feedforward postural adjustments following voluntary motor training Exp Brain Res 2007 181 537 546 10.1007/s00221-007-0950-z 17476489

[B26] Langer N Hanggi J Muller NA Simmen HP Jancke L Effects of limb immobilization on brain plasticity Neurology 2012 78 182 188 10.1212/WNL.0b013e31823fcd9c 22249495

